# Surveillance of pneumococcal serotype 1 carriage during an outbreak of serotype 1 invasive pneumococcal disease in central Australia 2010–2012

**DOI:** 10.1186/1471-2334-13-409

**Published:** 2013-09-03

**Authors:** Jana YR Lai, Heather Cook, Teem-Wing Yip, Jeanette Berthelsen, Stephen Gourley, Vicki Krause, Helen Smith, Amanda J Leach, Heidi Smith-Vaughan

**Affiliations:** 1 Menzies School of Health Research, Charles Darwin University, Darwin, NT, Australia; 2 Department of Health, Centre for Disease Control, Darwin, NT, Australia; 3 Flinders University Northern Territory Clinical School, Adelaide, NT, Australia; 4 Alice Springs Hospital Emergency Department, Alice Springs, NT, Australia; 5 Forensic & Scientific Services, Queensland Health, Brisbane, QLD, Australia

**Keywords:** Invasive pneumococcal disease, Serotype 1, Central Australia, Carriage

## Abstract

**Background:**

An outbreak of serotype 1 invasive pneumococcal disease (IPD) occurred in Central Australia from October 2010 to the latter part of 2012. Surveillance of serotype 1 carriage was conducted to determine epidemiological features of asymptomatic carriage that could potentially be driving the outbreak.

**Methods:**

130 patients and accompanying persons presenting at Alice Springs Hospital Emergency Department consented to nasopharyngeal swab (NPS) collection. NPS were processed by standard methods, including culture, pneumococcal *lyt*A quantitative real-time PCR, serotype 1-specific real-time PCR and multi-locus sequence typing (MLST).

**Results:**

Pneumococcal carriage was detected in 16% of participants. Carriage was highest in the under 10 year olds from remote communities surrounding Alice Springs (75%). Four NPS were positive for serotype 1 DNA by PCR; 3 were also culture-positive for serotype 1 pneumococci. Serotype 1 isolates had atypical colony morphology on primary culture. All serotype 1 carriers were healthy children 5 to 8 years of age from remote communities. By MLST, serotype 1 isolates were ST306, as were IPD isolates associated with this outbreak.

**Conclusions:**

During an outbreak of serotype 1 ST306 IPD, carriage of the outbreak strain was detected in 3% NPS collected. All carriers were healthy children 5 to 8 years of age.

## Background

Pneumococcal (*Streptococcus pneumoniae*) disease is a major contributor to morbidity and mortality worldwide [1]. The WHO estimates that pneumococcal disease is responsible for at least 1 million child deaths annually [2]. Greater than 90% of these deaths occur in developing countries where few children have access to pneumococcal vaccines [3].

More than 90 different pneumococcal serotypes exhibiting a wide range of epidemiological profiles have been reported [4]. Pneumococcus serotype 1 is one of the seven most common serotypes globally that cause IPD [3]. Serotype 1 is ranked among the top four serotypes in Africa, Asia and Latin America, which experience the highest IPD burden [3]. Serotype 1 is commonly associated with outbreaks of IPD and can cause severe disease including meningitis, complicated pneumonia and pulmonary empyema [5].

Certain serotypes are commonly found in the majority of nasopharyngeal carriage isolates from children [4]. Serotype 1 is rarely found in asymptomatic nasopharyngeal (NP) carriage, even in areas of high serotype 1 IPD [6,7]. This is believed to be a function of its low density and brief duration in the nasopharynx. During epidemics of serotype 1 IPD, the probability of detecting asymptomatic carriage is increased, as demonstrated by reports from populations including Belgium [8] and Portugal [9,10].

Young Indigenous children in remote communities in Australia have pneumococcal carriage rates of approximately 80%, and high rates of IPD, pneumonia and otitis media, related to endemic overcrowding [11]. In 2001, a 7-valent pneumococcal conjugate vaccine (PCV7) was included in the Northern Territory (NT) Childhood Vaccination Schedule for Indigenous and high-risk non-Indigenous infants as a 2, 4 and 6 month schedule with 23-valent pneumococcal polysaccharide vaccine (PPV23) recommended as the booster dose for Indigenous children at 18 months of age. PCV7 (2, 4, 6 months, with no booster) was introduced nationally for all infants in 2005. On 1st October 2009 the NT introduced 10-valent pneumococcal *Haemophilus influenzae* protein D conjugate vaccine (PHiD-CV10), offering additional protection against serotypes 1, 5 and 7F as a 2, 4, 6 and 18 month schedule for all children. Since 1999, PPV23, which offers protection against serotype 1, has been recommended for Indigenous adults who are 15 years of age or older, and in 2005 for all Australians 65 years of age or those medically at risk [12-14].

In Australia there have been documented cases of serotype 1 isolation from NP carriage samples [5,15,16]. During a serotype 1 IPD outbreak in 1991 in Central Australia, serotype 1 upper respiratory tract carriage was reported in 13 of 75 (17.3%) hospitalised children [15]. Serotype 1 NP carriage in Indigenous communities was also reported in northern regions of the Northern Territory in 1992–1993, 2002–2003, and 2005 [5,16]. These studies reported asymptomatic NP carriage of serotype 1 in children; the cases were not associated with serotype 1 IPD outbreaks. In the 2002 surveillance study, serotype 1 was carried by 13% (21/158) children under 11 years [5].

In November 2010, Alice Springs and surrounding areas, known as Central Australia, serviced by the Alice Springs Hospital, reported a 65% increase in IPD cases compared to the previous year [17]. IPD cases were primarily serotype 1 Sequence Type (ST) 306. The outbreak continued until the latter part of 2012. Through the peak until end of 2011, there had been a total of 69 cases of serotype 1 ST306 IPD, with 84% of cases Indigenous, median age 10 years (range 3–61 years) [17] and mean age 18 years (SD 16–21) (pers. Comm. H. Cook). A further 18 cases were identified in 2012 [17]. The aim of this study was to identify the potential serotype 1 carriage reservoir in the region.

## Methods

### Population

This study was in response to a request for surveillance of pneumococcal carriage in Central Australia during an outbreak of IPD. Central Australia has a geographical area of 830 000 square kilometres and a population of over 46 000, with a population of 28 000 in the town of Alice Springs. Alice Springs Hospital is the main hospital for Central Australia. The target population in this study was the residents of Central Australia.

### Collection and participation criteria

During March 18–21, 2011, nasopharyngeal swabs (NPS) were collected from consenting participants presenting to or visiting the Alice Springs Hospital Emergency Department. Those meeting the following criteria were excluded: Triage 1 and 2 patients, (requiring urgent or immediate care); apparently under the influence of alcohol or other drugs; having special needs; in police custody; and very recent arrivals (tourists) into Central Australia. Signed informed consent was obtained for collection of a single nasopharyngeal swab and for access to individual pneumococcal vaccination data. Consent included options for testing swabs for pneumococcus alone, and/or laboratory storage for future ethically approved studies of respiratory pathogens. Place of residence and date of birth were also collected. The Central Australian Human Research Ethics Committee provided a waiver for this surveillance study as it was in response to a disease outbreak requiring a timely response.

### Sample collection

Once consent was obtained, NPS were collected using rayon tipped swabs on flexible aluminium shafts. The quality of the sample was recorded as good (inserted into the nasopharynx for 5 seconds), fair (inserted briefly into the nasopharynx), poor (external nares) or sample collected with the swab after nose blowing into a tissue [18]. Nose blowing is not a standard sample collection method but offers an alternative specimen collection for children who refuse a nose swab and who have visible nasal discharge [18]. Swabs were immediately placed into 1ml of skim milk tryptone glucose glycerol broth (STGGB) [19] and frozen on dry ice. After each day of collection, frozen samples were transferred to a liquid nitrogen vapour shipper for storage and transport within 6 days to a −80°C freezer at the Menzies School of Health Research laboratory in Darwin NT, Australia.

### Pneumococcal culture

Following WHO guidelines for the routine laboratory culture of pneumococcus [19], 10 μl of the 1 ml NPS in STGGB were cultured and grown to determine the presence of pneumococcus by colony morphology, optochin susceptibility, the Phadebact© pneumococcus test and positive Quellung omniserum reaction. At least two presumptive pneumococcal colonies were selected for serotyping, including morphologically distinct colonies. Pneumococcal serotyping of isolates was by Quellung method [19]. Antibiotic susceptibility followed the calibrated dichotomous susceptibility (CDS) method for oxacillin (1 μg), penicillin (0.5 μg), tetracycline (30 μg), sulfamethoxazole/trimethoprim (25 μg), erythromycin (5 μg), chloramphenicol (30 μg) and azithromycin (15 μg) [20]. Isolates non-susceptible to penicillin or azithromycin had minimum inhibitory concentration (MIC) determined using E-tests (BioMerieux, France). Using the Clinical and Laboratory Standards Institute (CLSI) guidelines, penicillin breakpoints for intermediate resistance and resistance were > 0.06 ― ≤ 2 μg/ml and >2 μg/ml respectively [21]. Azithromycin breakpoints for intermediate resistance and resistance were > 0.25 - ≤ 0.5 μg/ml and > 0.5 μg/ml respectively [21]. Presumptive non-capsular pneumococci (NCSpn) were identified as optochin sensitive, Phadebact© pneumococcus test negative, bile soluble and Quellung Omni serum negative.

### DNA extraction from NPS samples

DNA was extracted from 100 μl aliquots of each NPS using a QIAamp 96 DNA blood kit (Qiagen, Germany) following enzymatic lysis as described previously [22]. The DNA was used in both the probe-based quantitative real-time PCR (qPCR) targeting *lytA* and the serotype 1 detection by real-time PCR as described below. For the qPCR 2 μl of sample was used in each reaction and for serotype 1 real-time PCR 1 μl of sample was used in each reaction.

### Pneumococcal detection and estimation of load by qPCR

Probe-based qPCR targeting *lytA* was used for detection of pneumococcus and estimation of pneumococcal load in NPS as previously described using forward (5′-TCTTACGCAATCTAGCAGATGAAGC-3′) and reverse (5′-GTTGTTTGGTTGGTTATTCGTGC-3′) primers and probe (5′- [6-FAM]-TTTGCCGAAAACGCTTGATACAGGG- [TAMRA]-3′), with product size of 101 bp [22,23]. All samples were run in duplicate in a Rotor-Gene 6000 real-time thermocycler (Corbett Research). Five pneumococcal DNA standards (reference strain ATCC49619) from 9 to 90,000 genome copies per reaction were included in each run along with multiple template controls of common respiratory pathogens. The limit of detection for this assay is 8.92 cells, equivalent to 4460 cells/ml. Successful qPCR assays had a standard curve correlation coefficient and efficiency of > 0.99 and > 0.86 respectively. All samples and standards were run in duplicate and required to amplify within 0.5 cycles. Any samples that did not fit the acceptability criteria were re-tested up to two times before being rejected and considered negative for pneumococcal DNA.

### Serotype 1 detection real-time PCR

For detection of serotype 1 from NPS samples, serotype 1 primers described for multiplex PCR serotyping by Pai et al. (2006), using forward (5′-CTCTATAGAATGGAGTATATAAACTATGGTTA-3′) and reverse (5′-CCAAAGAAAATACTAACATTATCACAATATTGGC-3′) primers, with product size of 280 bp [24] were used in a SYBR-based (Bioline, London) real-time PCR. For the positive control a high concentration (8.92 × 10^4^ copies/μl) and low concentration (8.92 copies/μl) of pneumococcal serotype 1 DNA was used. Cycling conditions were as follows; 50°C 2 mins, 95°C 10 mins, 45 cycles of 94°C for 30 s, 54°C for 30 s, 68°C for 60 s. A melt curve was generated for each reaction and any curve that deviated from the control melt curve was considered negative for serotype 1 DNA. Melt-curve analysis was done between 50-99°C, with 1°C each step with weight of 90 s pre-melt conditioning on first step and 5 s wait for each step thereafter. Both SYBR-based real-time and melt-curve analysis were done in a Rotor-Gene 6000 real-time thermocycler (Corbett Research).

### Serotype 1 multilocus sequence typing (MLST)

A single serotype 1 isolate from each of the serotype 1 culture positive NPS was analysed by MLST using modified primers as per Marsh et al. [21]. PCR products were sequenced, using the forward and reverse primers from each PCR (Macrogen, South Korea). Analysis of results was done using DNA Star (DNA star, USA); final sequences were submitted to the MLST database (http://www.mlst.net).

### Statistical analysis

All results were entered and analysis of results was done using STATA version 11.

## Results

In total, 130 people consented to participate in the study; approximately 40% of people approached refused to participate. The age range of the participants was 1–77 years (mean 37.2 years, SD ±20.5 years); 25% were 10 years of age and younger, 6% were 11–18 years of age, and 69% were 19–77 years. Twenty-seven participants were from 13 remote communities surrounding Alice Springs and 103 were from Alice Springs.

### Pneumococcal carriage

Twenty-one NPS (21/130, 16.2%) were positive by culture for capsular pneumococci and nineteen NPS (19/130, 14.6%) were positive for pneumococcal *lytA* DNA. By culture, carriage was highest (9/12, 75.0%) in the age group of 10 years and younger from remote communities, compared with 47.6% (10/21) of children in Alice Springs (Table 1). Pneumococcal carriage in the greater than 10 years age group was 0% (0/82) for Alice Springs residents, and 13.3% (2/15) for residents of remote communities.

**Table 1:** Demographic information of participants in study by age and residence

	**Number in study**	**Pneumococcus culture positive (95% CI)**	**Pneumococcus PCR positive (95% CI)**	**Pneumococcus serotype 1 culture positive (95% CI)**	**Pneumococcus serotype 1 PCR positive (95% CI)**
**Residence**					
Alice Springs	103/130 (79.2%)	10/103 (9.7%) (5, 17)	11/103 (10.7%) (5, 18)	0/103 (0.0%)	0/103 (0.0%)
Remote Communities	27/130 (20.8%)	11/27 (40.7%) (22, 61)	8/27 (29.6%) (14, 50)	3/27 (11.1%) (2, 29)	4/27 (14.8%) (4, 34)
**Alice Springs**					
≤10years	21/103 (20.4%)	10/21 (47.6%) (26, 70)	10/21 (47.6%) (26, 70)	0/21 (0.0%)	0/21 (0.0%)
>10years	82/103 (79.6%)	0/82 (0.0%)	1/82 (1.2%) (0.03, 7)	0/82 (0.0%)	0/82 (0.0%)
**Remote Communities**					
≤10years	12/27 (44.4%)	9/12 (75.0%) (43, 95)	8/12 (66.7%) (18,62)	3/12 (25.0%) (5, 57)	4/12 (33.3%) (10, 65)
>10years	15/27 (55.6%)	2/15 (13.3%) (2, 40)	0/15 (0.0%) (0, 22)	0/15 (0.0%)	0/15 (0.0%)

Data also include number of participants positive for pneumococcus by both culture and molecular methods.

### Pneumococcus serotype 1 carriage

Three samples were culture positive for serotype 1, with high semi-quantitative counts. In these samples, there was no detection of co-carriage with other serotypes following selection of two colonies and any that were morphologically distinct. On culture, serotype 1 isolates had an atypical morphology in that they were small and dry or large, crenelated and dry.

DNA extracts from all samples were screened for the presence of pneumococcus serotype 1 DNA. The three samples positive by culture for serotype 1 were also positive by PCR, and a further serotype 1 DNA positive sample. This sample was culture positive for 10A but not for serotype 1 despite screening 100 colonies on the plate using the Quellung reaction. All serotype 1 PCR positive samples were from children 5 to 8 years of age from three remote communities in Central Australia.

MLST analysis was undertaken on serotype 1 isolates acquired by microbiological culture from the three culture-positive children. The isolates had the same allelic profile by MLST, corresponding to sequence type (ST) 306.

### Pneumococcal conjugate vaccination status

Of the 130 participants sampled, there were 33 children who were 10 years or younger. Twenty-one children 10 years and younger had received a primary course of PCV7, with 16 of those also receiving a booster of PPV23. Four children had received a primary course of PHiD-CV and 8 children received a combination of PCV7 and PHiD-CV for their primary course. Two children did not provide consent or the data were missing. Of the four children positive for serotype 1 carriage, one did not provide consent to access their immunisation records, one had missing immunisation records and two had received a primary course of PCV7 with a PPV23 booster. The two adults positive for pneumococcal carriage both had received two doses of PPV23.

### Pneumococcal serotype, antimicrobial resistance, and load

**Figure 1:**
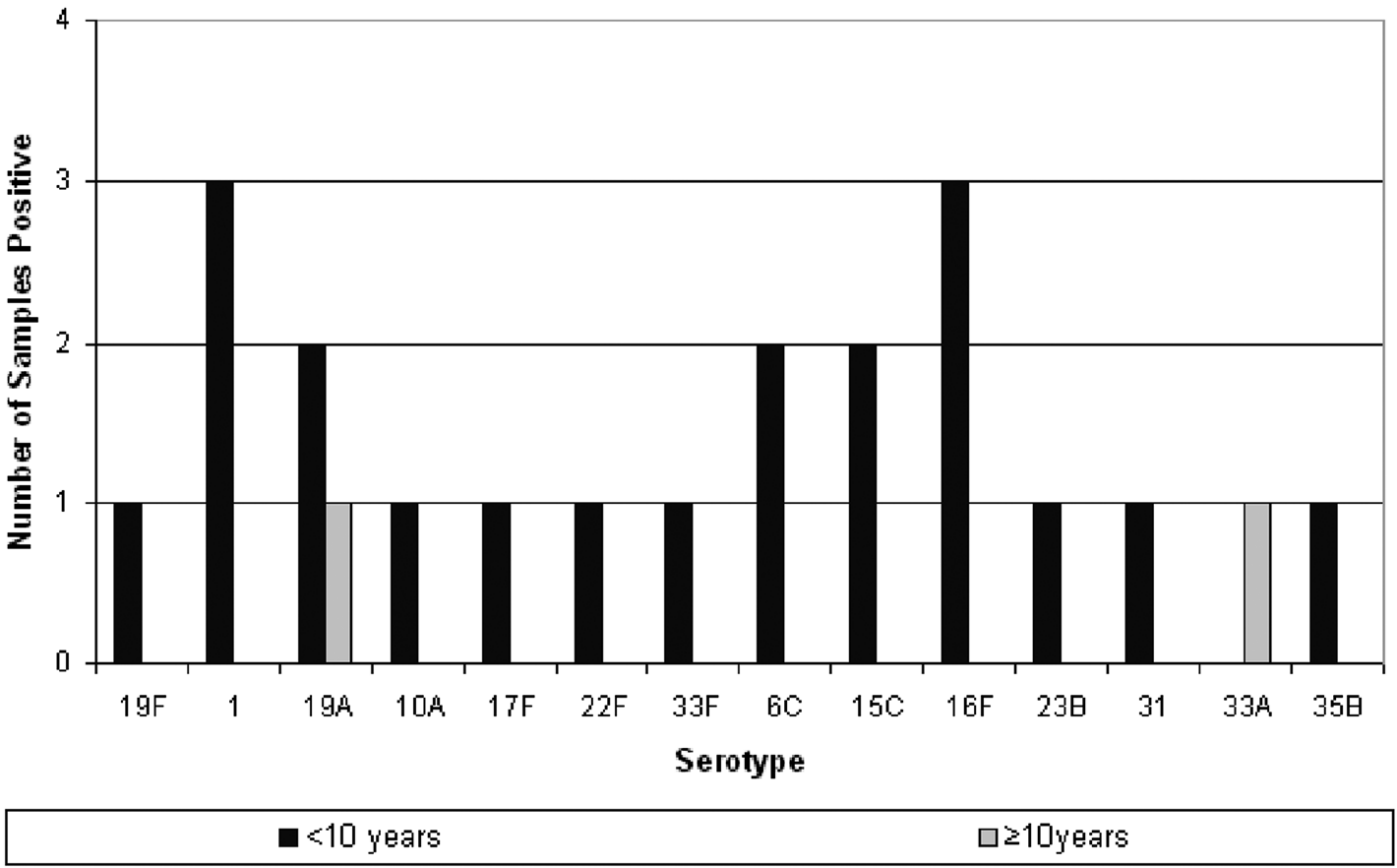
**Distribution of pneumococcal serotypes detected in culture, by age group; <10years and** ≥**10years.** PCV7 has serotypes 4, 6B, 9V, 14, 18C, 19F, and 23F (covers 4.8% positive swabs), PHiD-CV also has serotypes 1,5, 7F (covers 19.1% positive swabs), PCV13 also has serotypes 3, 6A, 19A, (covers 33.3% of positive swabs) and PPV23 has serotypes 1, 2, 3, 4, 5, 6B, 7F, 8, 9N, 9V, 10A, 11A, 12F, 14, 15B, 17F, 18C, 19A, 19F, 20, 22F, 23F and 33F (covers 52.4% of swabs).

Fifteen different serotypes of capsular pneumococci were identified from the 21 pneumococcus positive swabs (Figure 1). Multiple serotype carriage was observed in 1 swab, which had serotypes 15C and 35B.

Penicillin intermediate resistance and azithromycin resistance was detected in 7 of 22 (31.8%; serotypes 16F, 19A, 19F, 31) and 5 of 22 (22.7%; serotypes 16F, 17F, 19A, 22F, 31) isolates respectively. Serotype 1 isolates were susceptible to both antibiotics.

qPCR failed to detect 4 culture-positive swabs, while culture failed to detect 2 PCR-positive swabs. The serotypes that were detected by culture in these PCR-negative samples were 6C, 19A, 33A and 33F. The range of pneumococcal DNA concentration in the NPS samples detected by qPCR was 1.51x10^3^ to 7.15x10^6^ cells/ml.

### Non-capsular pneumococci (NCSpn)

Eight samples were positive by culture for NCSpn (6.1%), and co-carriage with capsular pneumococci was detected in 5 samples (serotypes 6C, 16F, 19F, 23B and 31). Seven of 8 NCSpn were non-susceptible to penicillin; six displayed intermediate resistance and one was resistant. Seven of eight NCSpn were resistant to azithromycin.

## Discussion

Pneumococcal carriage was detected in 16% (21/130) participants. Serotype 1 carriage was detected in four of 33 participants (12%) under the age of 10 years; three by culture and a fourth by real-time PCR. Overall pneumococcal carriage rates were also highest in this age group (19/33; 58%). Children from remote communities had the highest carriage at 75% (9/12). Of interest, all serotype 1 carriers were healthy children 5 to 8 years of age from remote communities. The three samples with culture-confirmed serotype 1 had high semi-quantitative counts and no co-colonisation with other serotypes detected.

Serotype 1 is included in the 10-valent pneumococcal conjugate vaccine, PHiD-CV, but not in PCV7. All participants who received at least one dose of PHiD-CV, had no serotype 1 carriage. Among the four participants positive for serotype 1, two had either missing immunisation records or no consent to access their records and the other two had received a full course of PCV7 with a booster of PPV23.

All serotype 1 isolates were both penicillin and azithromycin susceptible, whereas isolates of serotypes 16F, 17F, 19A, 19F, 22F, 31, and NCSpn displayed resistance to one or both of these antibiotics. All serotype 1 isolates from culture-positive samples were MLST ST306. IPD isolates associated with this outbreak were also ST306 [17]. This ST has been reported throughout Europe [6], from sterile and non-sterile sites.

The serotype 1 isolates detected in primary culture, and after our routine method of freezing in STGGB for transport and storage, had an atypical morphology. Serotype 1 isolates were small and dry or large, crenelated and dry, even though they were grown in a humid 37°C CO_2_ incubator. Upon passage, the colony morphology changed to a smooth, typical pneumococcal morphology. Serotype 1 is reported to be rarely detected in carriage, even in areas of high serotype 1 IPD [6,7]. This is primarily believed to be due to low density and short duration of colonisation [4]; however atypical morphology may lead to missed observations of carriage, and our finding further emphasises the importance of considering atypical pneumococcal colony morphologies in pneumococcal carriage studies.

In previous studies investigating serotype 1 carriage, there was limited documentation of co-colonisation with other serotypes; a single study reported 34% simultaneous colonisation with other pneumococcal serotypes [5]. In the current study, co-colonisation was not detected by selecting two colonies per culture plate and any that were morphologically distinct. The three NPS serotype 1 positive samples had three colonies each identified as serotype 1; however one of the serotype 1 DNA positive samples was positive in culture for serotype 10A. It is uncertain whether serotype 1 has a propensity to colonise with certain serotypes due to the limited data we have and also from the scarce literature available.

In this study there were more pneumococcal culture positive samples than pneumococcal qPCR positive samples. This discrepancy may be due to the lower limit of detection of culture (100 cells/ml) compared with qPCR (4460 cells/ml). We previously reported a higher sensitivity for PCR (likely due to detection of metabolically inactive cells), despite the disparity in limit of detection [22]. However, this was in high density carriage populations. In low density carriage populations the disparity in limit of detection may become more pronounced.

A limitation of this study was the low number of participants and the restriction to a single hospital emergency department. Despite this limitation our results were consistent with the age of past serotype 1 colonisation in this population, where serotype 1 colonisation was detected in children 12 years of age and younger [5,15]. A larger prevalence study including remote communities in the region may have allowed more definitive conclusions on the age of risk of colonisation with serotype 1, and improve data on the pneumococcal serotype distribution in this population. Longitudinal sampling would have provided a more comprehensive picture of the epidemiology of serotype 1 carriage during the outbreak, including the movement of serotype 1 through the communities.

## Conclusions

Nasopharyngeal swabbing during a Central Australia outbreak of serotype 1 IPD (mean age 18 years) identified asymptomatic carriers of serotype 1 as children under the age of 10 years living in remote communities. Serotype 1 carriage was not detected in PHiD-CV vaccinated participants. Serotype 1 isolates had atypical colony morphology on primary culture, which could result in missed identification. Because newer formulations of pneumococcal vaccines include serotype 1, populations that have received them may have a lower prevalence of asymptomatic carriage of pneumococcus serotype 1, resulting in a lower a lower incidence of serotype 1 IPD. It would be useful to conduct more extensive carriage studies to further understand the population biology of pneumococcus, which may be able to inform outbreak control strategies.

## Abbreviations

IPD: Invasive pneumococcal disease; NPS: Nasopharyngeal swab; MLST: Multi-locus sequence typing; NPS: Nasopharyngeal swabs; ST: Sequence type; PCV7: 7-valent pneumococcal conjugate vaccine; 23vPPV: 23-valent pneumococcal polysaccharide vaccine; STGGB: Skim milk tryptone glucose glycerol broth; MIC: Minimum inhibitory concentration; qPCR: quantitative real-time PCR; NCSpn: Non-capsular *S. pneumoniae*; DNA: Deoxyribose nucleic acid; WHO: World Health Organisation; SD: Standard deviation; PCR: Polymerase chain reaction; NT: Northern Territory of Australia; PHiD-CV10: 10-valent pneumococcal *Haemophilus influenzae* protein D conjugate vaccine.

## Competing interests

The authors declare that they have no competing interests.

## Authors’ contributions

Conception of study by AJL, HSV, HC, VK, TWY. AJL, HSV, JL study design. HSV, JL, TWY, JB, SG collection of samples. HS processed IPD samples and provided data for IPD isolates. All authors contributed to manuscript production. All authors read and approved the final manuscript.

## Authors’ information

A J Leach and H Smith-Vaughan equal authorship.

## Pre-publication history

The pre-publication history for this paper can be accessed here:

http://www.biomedcentral.com/1471-2334/13/409/prepub
